# RDA-MTE: an innovative model for emotion recognition in sports behavior decision-making

**DOI:** 10.3389/fnins.2024.1466013

**Published:** 2024-11-14

**Authors:** Sheng'ao Zhang

**Affiliations:** College of Physical Education, Huazhong University of Science and Technology, Wuhan, China

**Keywords:** emotional stimuli, motor behavior decision-making, emotion recognition, Multi-layer Transformer Encoder, ResNet-50, the bidirectional attention

## Abstract

Emotional stimuli play a crucial role in sports behavior decision-making as they significantly influence individuals' responses and decisions in sports contexts. However, existing research predominantly relies on traditional psychological and behavioral methods, lacking in-depth analysis of the complex relationship between emotions and sports behavior, particularly in the integration of real-time emotion recognition and sports behavior decision-making. To address this issue, we propose a deep learning-based model, RDA-MTE, which efficiently extracts and enhances feature interaction capabilities to capture and recognize facial expressions, thereby analyzing the impact of emotional stimuli on sports behavior decision-making. This model combines a pre-trained ResNet-50, a bidirectional attention mechanism, and a multi-layer Transformer encoder to improve the accuracy and robustness of emotion recognition. Experimental results demonstrate that the RDA-MTE model achieves an accuracy of 83.54% on the FER-2013 dataset and 88.9% on the CK+ dataset, particularly excelling in recognizing positive emotions such as “Happy” and “Surprise.” Additionally, the model exhibits strong stability in ablation experiments, validating its reliability and generalization capability across different emotion categories. This study not only extends research methodologies in the fields of affective computing and sports behavior decision-making but also provides significant reference for the development of emotion recognition systems in practical applications. The findings of this research will enhance understanding of the role of emotions in sports behavior and promote advancements in related fields.

## 1 Introduction

Emotion plays a critical role in human decision-making, particularly in sports, where emotional stimuli significantly impact athletes' decision-making processes. Emotional stimuli refer to emotional responses triggered by external environments or internal psychological states, such as anger, happiness, and fear (Robazza et al., 2022). These emotional responses affect athletes' reaction speed, judgment accuracy, and strategy choices during competitions. For instance, anger may lead to aggressive decisions, while fear may result in conservative strategies. However, current research on the relationship between emotional stimuli and sports decision-making faces many challenges (Niubò Solé et al., 2022). The diversity and complexity of emotions make their impact difficult to quantify and standardize. Additionally, real-time accurate detection and analysis of emotional changes remain a challenge.

Deep learning technology has made significant progress in the study of sports decision-making. Through large-scale data and complex models, deep learning can capture subtle differences and complex patterns in sports behavior, providing more precise behavior predictions and decision support. For example, convolutional neural networks (CNNs) and recurrent neural networks (RNNs) have been widely used in athlete action recognition and trajectory prediction (Wang T. Y. et al., 2023; Ramesh and Mahesh, 2022). Additionally, deep reinforcement learning (DRL) has been employed to optimize athletes' strategy choices, enhancing their performance in competitions (Tamminen and Watson, 2022). However, most of these studies focus on athletes' actions and strategies, with relatively little emphasis on the inclusion and analysis of emotional factors. The integration of emotion recognition and decision-making remains a challenging problem. Facial recognition is an important method for emotion recognition, allowing real-time acquisition of athletes' emotional states through the analysis of facial expressions (Rahimian et al., 2022). Advances in facial recognition technology in terms of accuracy and real-time processing make it significant in sports decision-making research. Facial expressions, as a crucial manifestation of emotions, can provide key emotional information for sports decision-making. For example, during competitions, real-time monitoring of athletes' facial expressions can help assess their emotional states and adjust training plans or competition strategies accordingly (Ding N. et al., 2022). Furthermore, combining deep learning with facial recognition technology can improve the accuracy of emotion recognition, providing more reliable data support for sports decision-making (Perolat et al., 2022). Therefore, facial recognition holds promising applications in studying the impact of emotional stimuli on sports decision-making. In summary, emotional stimuli have a profound impact on sports decision-making. However, current research faces numerous challenges in quantifying and standardizing the influence of emotions (Wang T. Y. et al., 2023). While deep learning technology has shown great potential in sports decision-making research, the incorporation and analysis of emotional factors remain insufficient (Ciaccioni et al., 2023). As an essential means of emotion recognition, facial recognition is significant in studying the impact of emotions on sports decision-making. By integrating deep learning with facial recognition, we can better understand and utilize emotional information, optimizing athletes' decision-making processes.

In recent years, many studies have explored the integration of emotion recognition and sports decision-making, achieving significant progress. Some of these studies have employed deep learning models to improve the accuracy and efficiency of emotion recognition and behavior prediction. A study proposed a convolutional neural network (CNN)-based emotion recognition model to detect athletes' emotional states during training and competitions (Jekauc et al., 2024). This model uses multiple convolutional layers to extract features from facial expressions and employs fully connected layers for emotion classification. The model was trained on standard emotion datasets and tested in actual sports scenarios. Although the model achieved high accuracy in emotion recognition, it performed poorly in handling real-time video streams, exhibiting latency issues. Another study developed an emotion recognition model combining recurrent neural networks (RNNs) and long short-term memory networks (LSTMs) (Liu et al., 2023). This model captures emotional changes in time series using RNNs and utilizes LSTMs to handle long-term dependencies, recognizing behavior patterns under different emotional states. The study trained and validated the model using datasets containing facial expressions and physiological signals. Despite its excellent performance in capturing emotional changes, the model's high computational complexity led to long training times, making it unsuitable for real-time applications. A different research proposed a multimodal emotion recognition model combining CNNs and multilayer perceptrons (MLPs) (Geetha et al., 2024). This model leverages CNNs to extract image features and uses MLPs to process emotion-related physiological signals. The study demonstrated that this multimodal approach improved emotion recognition accuracy, especially when combining visual and physiological data. However, the model's reliance on high-quality multimodal data posed challenges in data collection and synchronization, limiting its broad application. Another study introduced a Transformer-based emotion recognition model, utilizing multi-head attention mechanisms to capture complex relationships between emotional features (Tang et al., 2024). This model was trained on emotion recognition datasets and showed outstanding performance in various emotion recognition tasks. The Transformer model accelerated emotion recognition through parallel processing and enhanced its ability to handle large-scale data. However, the model required extensive training data and computational resources, making it difficult to deploy in resource-constrained real-world applications.

While these studies have made significant advances in emotion recognition and sports decision-making, several shortcomings remain. CNN-based models perform poorly in handling real-time video streams. RNN and LSTM models have high computational complexity, making them unsuitable for real-time applications. Multimodal approaches depend on high-quality data, limiting their widespread application, and Transformer models demand significant computational resources, making them challenging to deploy in real-world applications. These limitations indicate that the effectiveness and efficiency of current emotion recognition technologies in practical applications still need improvement. Therefore, this research aims to improve emotion recognition models by integrating pre-trained ResNet-50, dual-direction attention mechanisms, and multi-layer transformer encoders (MTE). By addressing the shortcomings of existing models, we hope to provide more accurate and efficient emotion recognition and decision support in sports behavior decision-making.

To address these limitations, this study proposes the RDA-MTE model, which integrates a pre-trained ResNet-50, dual-direction attention mechanisms, and a Multi-layer Transformer Encoder (MTE). The model is designed to improve the accuracy and robustness of emotion recognition, particularly in sports decision-making scenarios. The combination of these advanced components allows for more efficient feature extraction, enhanced feature interaction, and improved handling of complex emotional states. We aim to improve emotion recognition, providing reliable emotional data for sports behavior decision-making, even when faced with diverse and challenging scenarios. The RDA-MTE model offers notable improvements in both accuracy and robustness for emotion recognition in sports scenarios. Its architecture enables efficient handling of complex emotional states while maintaining resource efficiency, making it highly adaptable to diverse and challenging environments. The model's ability to manage long-range dependencies and enhance feature interactions contributes to its reliability in supporting sports behavior decision-making.

Based on our research, we have made the following major contributions:

- We propose a novel emotion recognition network, RDA-MTE, which combines a pre-trained ResNet-50, a dual-direction attention mechanism, and a Multi-layer Transformer Encoder (MTE). This model significantly improves the accuracy and real-time performance of emotion recognition with limited data and computational resources. It provides an efficient solution for emotion recognition research, addressing the deficiencies of existing models in handling complex emotional states.
- By introducing the dual-direction attention mechanism, we enhance the interaction between features, enabling the model to perform excellently in processing complex emotional states. This enhancement enhances both the accuracy and robustness of emotion recognition, while also introducing new insights and methodologies for related research.
- Our research demonstrates the broad application prospects of RDA-MTE in practical scenarios, particularly in sports behavior decision-making. By providing accurate emotional data support, our model can help athletes and coaches better understand and manage emotions, thereby optimizing training and competition strategies and improving sports performance.

## 2 Related work

### 2.1 Transformers in computer vision

The Transformer architecture, initially successful in natural language processing, was quickly adopted in the field of computer vision. Transformers rely on multi-head attention mechanisms and parallel processing capabilities, excelling at capturing complex relationships between features, particularly in large-scale data processing and long-distance dependency capture (Parvaiz et al., 2023). Transformers significantly improved the performance of visual tasks through the self-attention mechanism and fully parallel processing! (Park and Kim, 2022). Researchers proposed the Vision Transformer (ViT), which treats an image as a sequence of image patches and serializes these patches into input tokens, allowing the application of the Transformer's self-attention mechanism (Touvron et al., 2022). The performance of ViT on the ImageNet dataset demonstrated the potential of Transformers in visual tasks. Further studies showed that the application of Transformers in visual tasks could be enhanced by introducing hierarchical window attention mechanisms, improving the model's efficiency and scalability, and addressing the computational complexity issues when processing high-resolution images (Han et al., 2022). These improved Transformer models achieved outstanding performance in various computer vision tasks, such as object detection and image segmentation.

In the field of emotion recognition, researchers have begun exploring the application of Transformer models. Transformer-based emotion recognition models use multi-head attention mechanisms to capture the complex relationships between facial expression features. Compared to traditional convolutional neural networks (CNNs), Transformers offer better global feature extraction capabilities (Li et al., 2023). Specifically, the multi-head attention mechanism in the Transformer architecture can concurrently attend to various segments of the input data, capturing both global and local emotional features (Ding M. et al., 2022). This capability is especially important when dealing with high-dimensional data such as images and videos, as it can more effectively integrate information and recognize complex emotional states. Transformer models have also been applied to emotion recognition in sports contexts. These models help capture subtle facial expressions and emotional responses to stress and exertion during sports activities such as training and competitions (Mekruksavanich and Jitpattanakul, 2022). By analyzing emotions like anxiety and motivation, they contribute to performance analysis and decision-making systems for athletes (Ramzan and Dawn, 2023).

Experiments have shown that Transformer models perform excellently in various emotion recognition tasks, surpassing traditional CNN models in accuracy and significantly improving processing speed (Tang et al., 2022). This is mainly due to the parallel processing capability of Transformer models, allowing them to handle large amounts of data in a relatively short time (Pan et al., 2022). However, Transformer models in emotion recognition applications also face some challenges. First, the Transformer architecture requires a large amount of training data to optimize model parameters and ensure performance in practical applications (Wu et al., 2022). For emotion recognition tasks, obtaining sufficiently large and accurately labeled emotion datasets is a difficult task. Second, the computational complexity of Transformer models is high, requiring substantial hardware resources, which limits their application in resource-constrained environments (Wang et al., 2022). Additionally, Transformer models need further optimization to improve adaptability to real-time video streams when dealing with dynamic emotional changes.

Despite these challenges, the application prospects of Transformer architectures in emotion recognition are broad. Their powerful feature extraction and parallel processing capabilities provide significant advantages in handling complex emotional features and large-scale data (Cao et al., 2022). With advancements in hardware technology and the enrichment of emotion datasets, Transformer-based emotion recognition models are expected to play a more significant role in practical applications.

### 2.2 Multimodal emotion recognition

Multimodal emotion recognition enhances accuracy and robustness by integrating data from multiple sources. These systems typically combine visual, audio, and physiological signals to capture more comprehensive and detailed emotional features (Zhang et al., 2023b). This method overcomes the limitations of single-modality emotion recognition approaches by leveraging information from different modalities, thus improving the model's performance in various complex scenarios (Pan et al., 2023). In the domain of sports, multimodal emotion recognition has shown great potential. Studies have combined facial expression analysis with physiological signals, such as heart rate and skin conductance, to monitor athletes' emotional responses during high-pressure situations (Zhou et al., 2020). For instance, the fusion of visual features with heart rate variability helps track emotions like fear and anxiety during physical exertion, offering insights into how these emotions influence athletic performance (Shoumy et al., 2020). This multimodal approach is particularly effective in dynamic, real-time sports environments.

Multimodal emotion recognition research has made significant progress. For example, the visual modality primarily includes facial expressions and eye movement features, while the audio modality encompasses characteristics such as the frequency, pitch, and rhythm of speech (Ahmed et al., 2023). By integrating these two modalities, it is possible to capture emotional changes more comprehensively. When a person is speaking, analyzing both their vocal characteristics and facial expressions can lead to a more accurate determination of their emotional state. Studies have shown that multimodal emotion recognition systems that combine visual and audio features perform excellently in handling different emotional states, particularly in recognizing subtle emotional changes and complex emotional expressions (Chen et al., 2022).

The fusion of visual and physiological signals is another crucial direction in multimodal emotion recognition. Physiological signals include heart rate, galvanic skin response (GSR), and electroencephalography (EEG) (Wang S. et al., 2023). These physiological signals exhibit significant changes with emotional states. By combining these signals with visual features such as facial expressions, the accuracy of emotion recognition can be further enhanced. For instance, when a person is nervous or anxious, their heart rate and GSR increase, and these changes can be combined with facial expression features to provide more comprehensive emotion recognition information (Le et al., 2023). Research has found that the integration of visual and physiological signals has significant advantages in detecting latent emotional states and complex emotional reactions. In multimodal emotion recognition methods, feature-level fusion involves integrating features from various modalities during the extraction stage to create a unified feature representation (Garcia-Garcia et al., 2023). This method includes techniques such as feature concatenation, feature weighted averaging, and principal component analysis (PCA). By concatenating visual and audio feature vectors, a high-dimensional feature vector can be formed, enhancing the feature representation capacity (Sharafi et al., 2022). Decision-level fusion involves combining the outputs of multiple classifiers during the classification stage to obtain the final recognition result. Common methods include voting, weighted voting, and Bayesian inference. By combining predictions from multiple classifiers, we can enhance the precision and reliability of emotion recognition, particularly in addressing complex recognition tasks. Hybrid fusion methods combine the advantages of both feature-level and decision-level fusion (Zhang et al., 2023a). Feature-level fusion can be performed during the feature extraction stage to form a comprehensive feature representation, followed by decision-level fusion of the outputs of multiple classifiers during the classification stage (Zhao et al., 2022). This approach can fully utilize the advantages of various fusion techniques, further improving recognition performance.

Multimodal emotion recognition offers significant advantages in many aspects. Firstly, multimodal fusion can effectively improve the accuracy of emotion recognition (Mocanu et al., 2023). For example, combining visual and audio features can maintain high recognition rates under various environmental conditions. Secondly, multimodal fusion can enhance the model's robustness in handling complex environments and varying conditions by combining different types of features. For instance, multimodal fusion can maintain high recognition performance under changes in lighting, background interference, and variations in emotional expression (Yoon, 2022). Finally, multimodal fusion techniques compress high-dimensional data while preserving feature representativeness and discriminative power, thereby enhancing model processing efficiency.

However, multimodal emotion recognition also faces some challenges. Firstly, collecting and synchronizing multimodal data is both complex and costly. Different modalities of data need to be collected at the same time and accurately synchronized, posing high demands on data collection equipment and technology (Ma et al., 2023). Secondly, processing multimodal data requires higher computational resources, increasing the system's complexity and the difficulty of real-time processing. To address these challenges, researchers continuously optimize multimodal fusion algorithms and model structures, exploring techniques such as data augmentation and transfer learning to enhance the model's performance in practical applications. Overall, multimodal emotion recognition significantly enhances accuracy and robustness by integrating features from diverse sources and types. With continuous technological advancements, multimodal emotion recognition methods are expected to demonstrate their vast potential in more practical applications.

## 3 Method

### 3.1 Overview of our network

**Figure 1:**
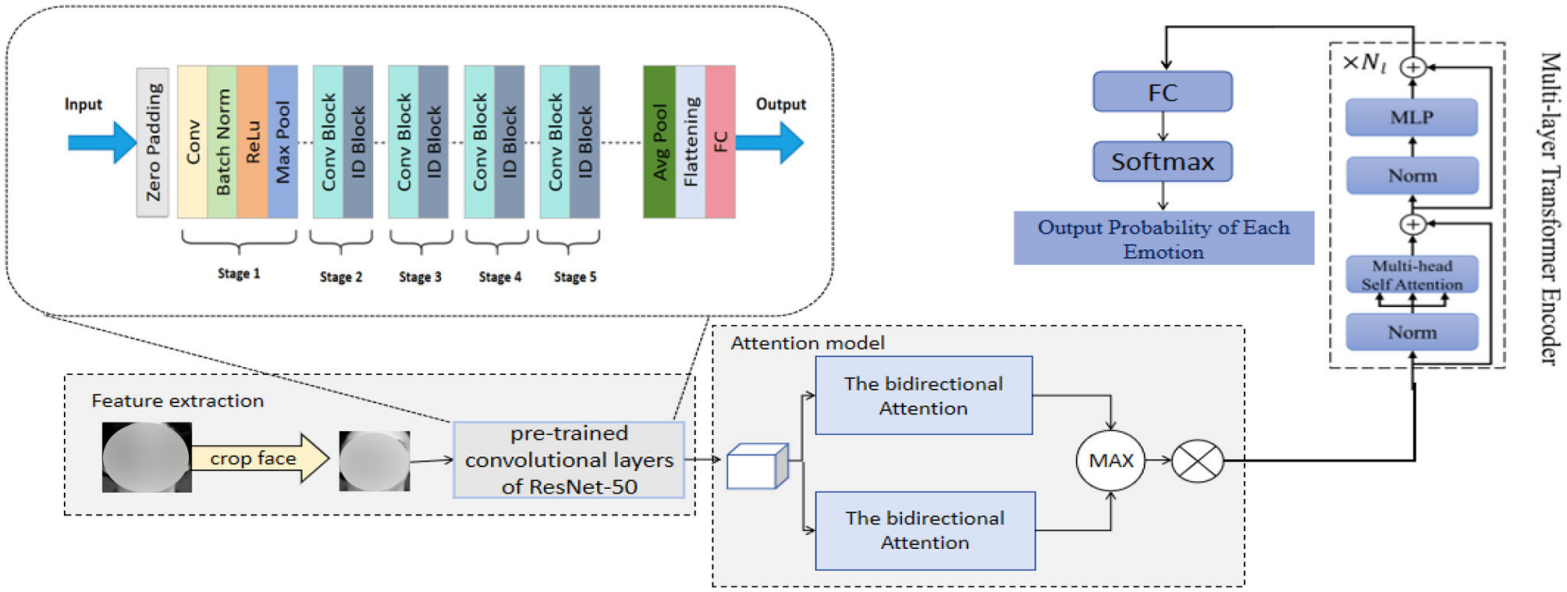
The overall structure of the RDA-MTE model.

We propose a novel emotion recognition network, RDA-MTE, which combines a pre-trained ResNet-50, bidirectional attention mechanism, and a multi-layer transformer encoder (MTE) to enhance the accuracy and real-time performance of emotion recognition. This network provides reliable emotional data support for sports behavior decision-making. ResNet-50, as a feature extractor, leverages its pre-trained convolutional layers to effectively capture subtle features in facial expressions. The primary role of this component is to extract high-quality visual features initially while reducing the training time and computational resource requirements. Building on the features extracted by ResNet-50, we introduce a bidirectional attention mechanism. This mechanism enhances the interaction between features by computing the global dependencies of the input features, thereby better capturing the complex relationships between facial expression features and improving the accuracy and robustness of emotion recognition. The MTE further encodes the features processed by the bidirectional attention mechanism, capturing long-distance dependencies. The transformer model has significant advantages in capturing long-distance dependencies and processing sequential data. Through the attention mechanism, it can process large-scale data in parallel, improving the efficiency and accuracy of emotion recognition. The incorporation of the multi-layer transformer encoder enables RDA-MTE to effectively handle complex emotional features and excel in emotion recognition tasks. The construction process of our RDA-MTE network is as follows: First, the pre-trained ResNet-50 is used to extract high-quality visual features from the input facial expression images. Each convolutional layer of ResNet-50 can capture feature information at different levels, ultimately resulting in a rich feature representation. Based on the features extracted by ResNet-50, a bidirectional attention mechanism is applied. By computing the global dependencies of the input features, the bidirectional attention mechanism can simultaneously focus on local and global information, enhancing the interaction between features. Finally, the features processed by the bidirectional attention mechanism are input into the multi-layer transformer encoder. The transformer model captures long-distance dependencies through the multi-head attention mechanism and further encodes the features. The introduction of MTE allows the model to process large-scale data in parallel, enhancing processing efficiency and accuracy. The features encoded by MTE are then input into a fully connected layer for emotion classification. The classifier learns the distinguishing information of different emotional features and ultimately outputs the emotion recognition results. Figure 1 illustrates the overall structure of our RDA-MTE model.

The application of the RDA-MTE model in emotion recognition is of great significance, especially in the field of sports behavior decision-making. Emotions directly affect the behavioral decisions of athletes. Real-time and accurate recognition of athletes' emotional states can provide valuable data support for coaches and athletes, helping to formulate training plans and competition strategies. By recognizing athletes' emotional states, training intensity and methods can be adjusted in a timely manner, avoiding the impact of emotional fluctuations on training effects. In competitions, real-time emotion recognition can help coaches adjust tactics based on athletes' emotional states, thereby increasing the probability of winning. Additionally, long-term monitoring of athletes' emotional changes can facilitate emotional intervention and management, improving psychological resilience and performance. In summary, the RDA-MTE model enhances the accuracy and real-time performance of emotion recognition, providing scientific support for sports behavior decision-making and contributing to the overall improvement of training effectiveness and competition results.

### 3.2 ResNet-50 feature extractor

**Figure 2:**
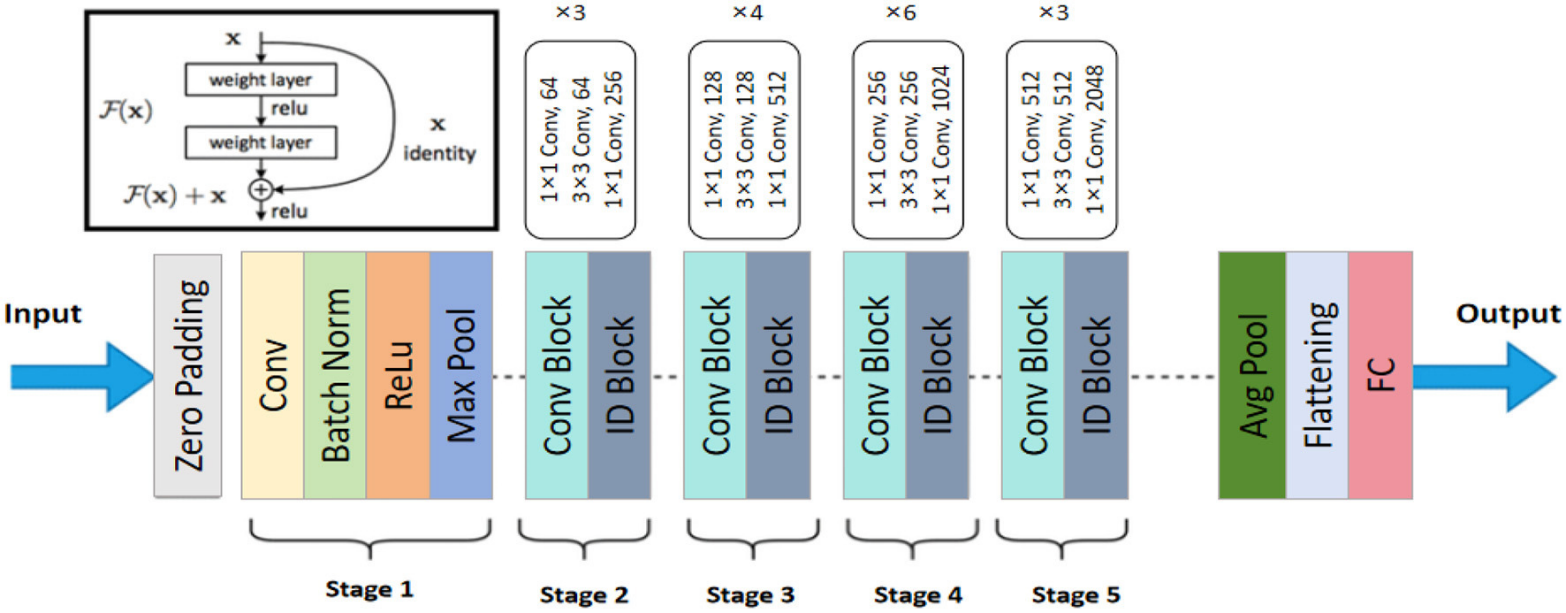
The architecture of the ResNet-50 used in the RDA-MTE model. The network consists of an initial convolutional layer followed by batch normalization and ReLU activation. It includes five stages of convolutional (Conv) and identity (ID) blocks, with each stage having varying numbers of blocks and convolutional filters. The final layers include an average pooling (Avg Pool) layer and a fully connected (FC) layer, producing the output features used for emotion recognition.

ResNet-50 is a deep convolutional neural network model that addresses the issues of vanishing and exploding gradients in the training of deep neural networks by introducing residual blocks. The core idea behind ResNet-50 is the use of skip connections, which allow input information to bypass one or more layers of the neural network and be directly transmitted to the output (Tian et al., 2022). This preserves the original information and accelerates the training process. Comprising 50 layers, ResNet-50 has a strong feature extraction capability, effectively capturing both fine details and high-level semantic information in images. Figure 2 shows the structure of ResNet-50. In the model architecture diagram, ResNet-50 consists of multiple residual blocks, each containing several convolutional layers and skip connections. This design enables ResNet-50 to maintain high feature extraction efficiency while avoiding the vanishing gradient problem, ensuring effective training of deep networks.

**Figure 3:**
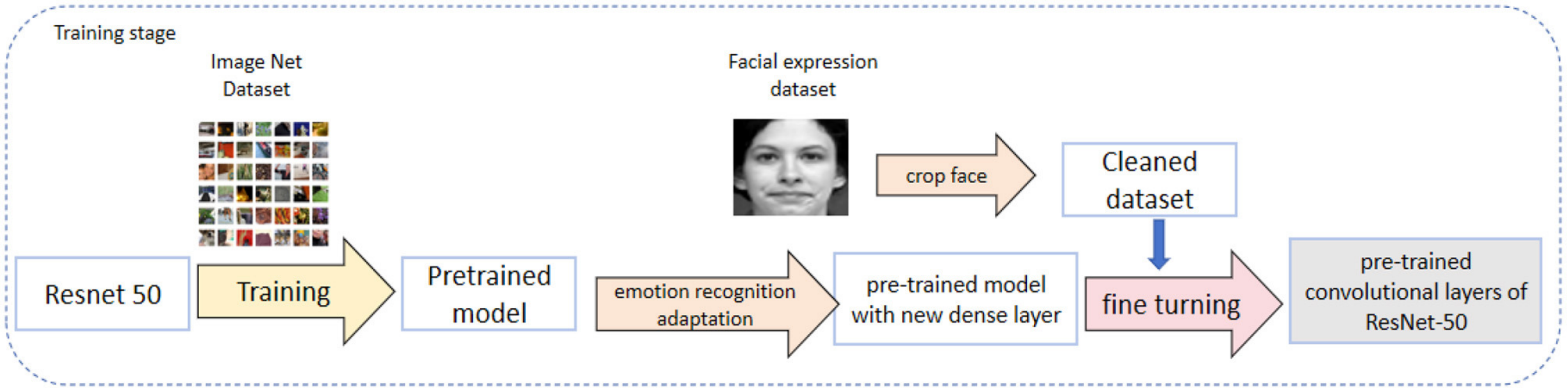
The pre-training process of ResNet-50. The process starts with training the ResNet-50 model on the ImageNet dataset to create a pre-trained model. This pre-trained model is then adapted for emotion recognition by adding a new dense layer and fine-tuning it using a cleaned facial expression dataset. The final result is a model with pre-trained convolutional layers of ResNet-50, fine-tuned for emotion recognition tasks.

In our RDA-MTE model, ResNet-50 serves as the feature extractor, playing a crucial role. First, by utilizing the pre-trained convolutional layers of ResNet-50, we can extract high-quality visual features from the input facial expression images. These features include the geometric structure of the face, texture details, and variations in lighting, providing a rich feature representation for subsequent processing. The pre-training process involves training ResNet-50 on a large-scale dataset, such as ImageNet, which contains millions of labeled images across thousands of categories. This extensive pre-training allows the model to learn a wide variety of visual features that are transferable to other tasks, such as facial expression recognition. Figure 3 illustrates the pre-training process of ResNet-50 on ImageNet.

Second, the pre-trained ResNet-50 model significantly reduces training time and computational resource requirements, improving the initial performance and stability of the model. This approach allows us to quickly and accurately extract essential features from facial expressions, laying a solid foundation for subsequent emotion recognition. By leveraging the knowledge gained during the pre-training phase, our RDA-MTE model benefits from enhanced feature extraction capabilities, leading to more accurate and robust emotion recognition results.

Here are the core mathematical formulations for ResNet-50:

Residual block:


(1) $$\begin{array}{l} \;x_{l+1}=F\left(x_{l},\left\{W_{l}\right\}\right)+x_{l} \end{array}$$

where *x*_*l*_ is the input to the *l*-th layer, $F\left(x_{l},\left\{W_{l}\right\}\right)$ is the residual mapping to be learned, and *W*_*l*_ are the weights of the *l*-th layer.

Residual mapping:


(2) $$\begin{array}{l} \;F\left(x_{l},\left\{W_{l}\right\}\right)=W_{l,2}\sigma \left(W_{l,1}x_{l}\right) \end{array}$$

where *W*_*l*, 1_ and *W*_*l*, 2_ are the weights of the *l*-th layer, and σ denotes the ReLU activation function.

Output of residual block:


(3) $$\begin{array}{l} \;y=W_{out}F\left(x_{l},\left\{W_{l}\right\}\right)+b_{out} \end{array}$$

where *y* is the output of the network, *W*_*out*_ are the weights of the output layer, and *b*_*out*_ is the bias of the output layer.

Loss function:


(4) $$L=\frac{1}{N}\sum_{i=1}^{N}\ell (y_{i},{\hat{y}}_{i})$$

where *L* is the loss function, *N* is the number of samples, *y*_*i*_ is the predicted value, ŷ_*i*_ is the ground truth, and ℓ is the loss for each sample.

Binary cross-entropy loss:


(5) $$\ell (y_{i},{\hat{y}}_{i})=-\left({\hat{y}}_{i}\log(y_{i})+(1-{\hat{y}}_{i})\log(1-y_{i})\right)$$

where ℓ(*y*_*i*_, ŷ_*i*_) is the binary cross-entropy loss for sample *i*.

Gradient descent update:


(6) $$\begin{array}{l} \;W_{l}\leftarrow W_{l}-\eta \frac{\partial L}{\partial W_{l}} \end{array}$$

where *W*_*l*_ are the weights of layer *l*, η is the learning rate, and $\frac{\partial L}{\partial W_{l}}$ is the gradient of the loss with respect to the weights.

Batch normalization and activation:


(7) $$\begin{array}{l} \;x_{l+1}=\sigma \left(BN\left(F\left(x_{l},\left\{W_{l}\right\}\right)+x_{l}\right)\right) \end{array}$$

where *BN* denotes the batch normalization function applied to the output of the residual block, and σ is the activation function.

Emotion recognition is a crucial prerequisite for implementing sports behavior decision-making. By accurately identifying the emotional states of athletes, valuable data support can be provided to coaches and athletes, helping them to better formulate training plans and competition strategies. The powerful feature extraction capability of ResNet-50 allows our RDA-MTE model to efficiently and accurately extract facial expression features, thereby enhancing the accuracy and real-time performance of emotion recognition. This improvement not only performs excellently in laboratory environments but also provides scientific evidence for sports behavior decision-making in practical applications, optimizing training effects and competition outcomes. In summary, the ResNet-50 feature extractor plays a pivotal role in our model by efficiently extracting facial expression features, significantly improving the accuracy and real-time performance of emotion recognition, and providing reliable data support for sports behavior decision-making. The application of this technology not only enhances the performance of emotion recognition models but also lays the foundation for achieving more scientific and precise sports behavior decision-making.

### 3.3 The bidirectional attention

**Figure 4:**
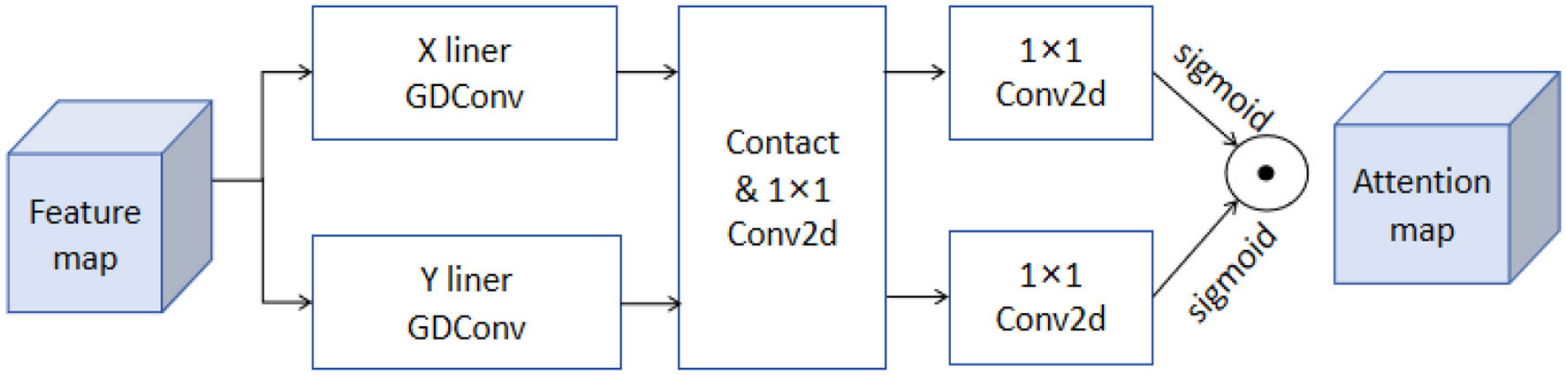
The architecture of the bidirectional attention mechanism. The mechanism starts with the feature map, which undergoes X and Y linear GDConv operations. The outputs are concatenated and processed through a 1 × 1 Conv2d layer. This is followed by two separate 1 × 1 Conv2d layers, with sigmoid activations, generating the attention map. This process enhances the interaction between features by computing global dependencies of the input features.

The bidirectional attention mechanism is a method for enhancing the information processing capability of neural networks by calculating the interdependencies among elements in the input sequence, allowing the model to capture both global and local information more effectively (Feng et al., 2022). In traditional unidirectional attention mechanisms, attention weights consider information from only one direction. In contrast, the bidirectional attention mechanism computes attention weights in both directions, enhancing the model's understanding of the structure and features of the input data. Figure 4 illustrates the structure of the bidirectional attention mechanism. In the model architecture diagram, it can be seen that the input features are processed through attention calculations in both forward and backward directions to obtain attention weights. These weights are then used to weight the input features, resulting in enhanced feature representations.

In our RDA-MTE model, the introduction of the bidirectional attention mechanism makes significant contributions. First, by calculating the global dependencies of the input features, the bidirectional attention mechanism enhances the interaction between features. This mechanism better captures the complex relationships among facial expression features, improving the accuracy and robustness of emotion recognition. Specifically, the bidirectional attention mechanism simultaneously focuses on both local and global information in facial expressions, enabling the model to perform better in handling complex emotional states. For instance, when input features are processed through the bidirectional attention mechanism, the model can recognize the importance of local regions such as the eyes and mouth and relate these local features to the overall facial expression, thereby forming a more comprehensive and accurate representation of emotions. The bidirectional attention mechanism, by incorporating attention calculations in both forward and backward directions, allows the model to infer the importance of subsequent features from current features and to trace back the importance of current features from subsequent features. This approach captures richer emotional feature information.

Here are the core mathematical formulations for the bidirectional attention Attention mechanism:

Forward attention scores:


(8) $$\begin{array}{l} \;e_{ij}^{\left(f\right)}={\text{w}}^{\left(f\right)⊤}\tanh\left({\text{W}}^{\left(f\right)}x_{i}+{\text{U}}^{\left(f\right)}h_{j-1}^{\left(f\right)}+b^{\left(f\right)}\right) \end{array}$$

where $e_{ij}^{\left(f\right)}$ is the forward attention score, **w**^(*f*)^, **W**^(*f*)^, and **U**^(*f*)^ are learnable parameters, *x*_*i*_ is the input feature, $h_{j-1}^{\left(f\right)}$ is the previous hidden state, and *b*^(*f*)^ is the bias term.

Backward attention scores:


(9) $$\begin{array}{l} \;e_{ij}^{\left(b\right)}={\text{w}}^{\left(b\right)⊤}\tanh\left({\text{W}}^{\left(b\right)}x_{i}+{\text{U}}^{\left(b\right)}h_{j+1}^{\left(b\right)}+b^{\left(b\right)}\right) \end{array}$$

where $e_{ij}^{\left(b\right)}$ is the backward attention score, **w**^(*b*)^, **W**^(*b*)^, and **U**^(*b*)^ are learnable parameters, *x*_*i*_ is the input feature, $h_{j+1}^{\left(b\right)}$ is the next hidden state, and *b*^(*b*)^ is the bias term.

Forward attention weights:


(10) $$\begin{array}{l} \;\alpha_{ij}^{\left(f\right)}=\frac{\exp\left(e_{ij}^{\left(f\right)}\right)}{\sum_{k=1}^{T}\exp\left(e_{ik}^{\left(f\right)}\right)} \end{array}$$

where $\alpha_{ij}^{\left(f\right)}$ is the forward attention weight, $e_{ij}^{\left(f\right)}$ is the forward attention score, and *T* is the length of the input sequence.

Backward attention weights:


(11) $$\begin{array}{l} \;\alpha_{ij}^{\left(b\right)}=\frac{\exp\left(e_{ij}^{\left(b\right)}\right)}{\sum_{k=1}^{T}\exp\left(e_{ik}^{\left(b\right)}\right)} \end{array}$$

where $\alpha_{ij}^{\left(b\right)}$ is the backward attention weight, $e_{ij}^{\left(b\right)}$ is the backward attention score, and *T* is the length of the input sequence.

Context vector:


(12) $$\begin{array}{l} \;c_{i}=\overset{T}{\underset{j=1}{\sum }}\left(\alpha_{ij}^{\left(f\right)}h_{j}^{\left(f\right)}+\alpha_{ij}^{\left(b\right)}h_{j}^{\left(b\right)}\right) \end{array}$$

where *c*_*i*_ is the context vector for the input element *x*_*i*_, $\alpha_{ij}^{\left(f\right)}$ and $\alpha_{ij}^{\left(b\right)}$ are the forward and backward attention weights, respectively, and $h_{j}^{\left(f\right)}$ and $h_{j}^{\left(b\right)}$ are the hidden states in the forward and backward directions.

The advantages of the bidirectional attention mechanism in capturing complex emotional features enable our RDA-MTE model to efficiently and accurately recognize athletes' emotional states, thereby enhancing the accuracy and real-time performance of emotion recognition. This improvement not only excels in laboratory environments but also significantly enhances sports behavior decision-making in practical applications, optimizing training effects and competition results. Overall, the bidirectional attention mechanism plays a crucial role in our model by enhancing the interaction between features, significantly improving the accuracy and robustness of emotion recognition. The application of this technology not only boosts the performance of emotion recognition models but also lays the foundation for achieving more scientific and precise sports behavior decision-making.

### 3.4 Multi-layer Transformer Encoder

**Figure 5:**
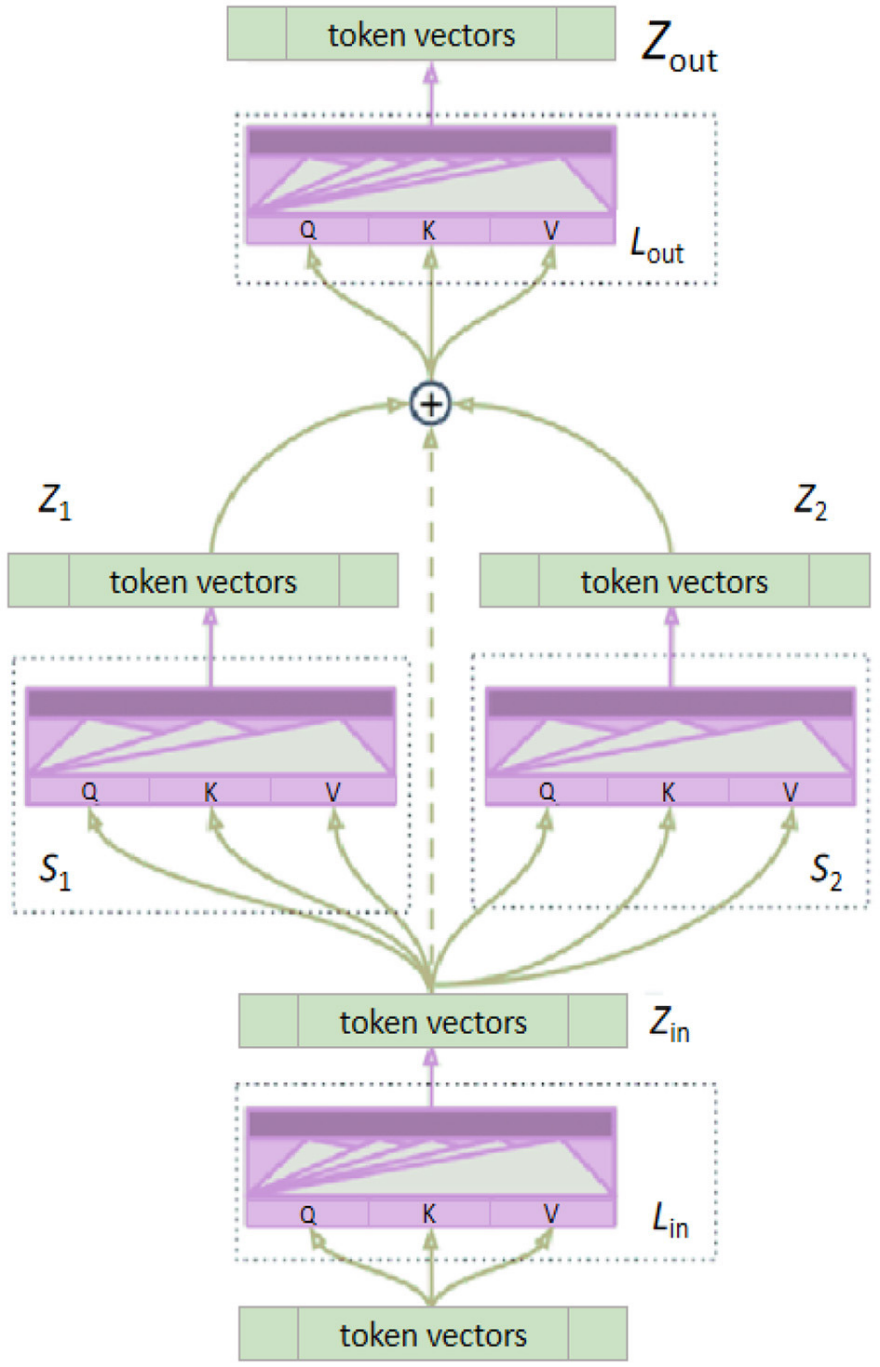
The architecture of the Multi-layer Transformer Encoder.

The Multi-layer Transformer Encoder (MTE) is a deep neural network model based on the self-attention mechanism, initially applied to natural language processing tasks. Its core concept is to capture long-range dependencies and global contextual information in input sequences through the stacking of multi-head self-attention mechanisms and feed-forward neural network layers (Yang et al., 2022). The multi-layer transformer encoder consists of several identical encoder layers stacked together. Each encoder layer comprises two main components: the multi-head self-attention mechanism and the feed-forward neural network. The multi-head self-attention mechanism calculates the attention weights of each element in the input sequence in parallel through multiple self-attention heads. The results from each attention head are then concatenated and linearly transformed to capture the global information from different positions in the input sequence, thereby enhancing the model's ability to handle long-range dependencies. The feed-forward neural network consists of two linear transformations and an activation function, used for further nonlinear transformation and feature extraction of the features processed by the self-attention mechanism. Each encoder layer also includes residual connections and layer normalization to ensure training stability and accelerate convergence. Figure 5 provides an overview of the Multi-layer Transformer Encoder architecture.

Here are five key mathematical formulations for MTE:

Self-attention:


(13) $$\begin{array}{l} \;\text{Attention}\left(Q,K,V\right)=\text{softmax}\left(\frac{QK^{T}}{\sqrt{d_{k}}}\right)V \end{array}$$

where *Q* is the query matrix, *K* is the key matrix, *V* is the value matrix, and *d*_*k*_ is the dimensionality of the keys.

Multi-head attention:


(14) $$\begin{array}{l} \;\text{MultiHead}\left(Q,K,V\right)=\text{Concat}\left({\text{head}}_{1},\ldots ,{\text{head}}_{h}\right)W^{O} \end{array}$$

where ${\text{head}}_{i}=\text{Attention}\left(QW_{i}^{Q},KW_{i}^{K},VW_{i}^{V}\right)$, $W_{i}^{Q},W_{i}^{K},W_{i}^{V}$ are the projection matrices for the *i*-th head, and *W*^*O*^ is the output projection matrix.

Position-wise feed-forward network:


(15) $$\begin{array}{l} \;\text{FFN}\left(x\right)=\max\left(0,xW_{1}+b_{1}\right)W_{2}+b_{2} \end{array}$$

where *W*_1_ and *W*_2_ are weight matrices, *b*_1_ and *b*_2_ are bias terms, and max(0, ·) denotes the ReLU activation function.

Layer normalization:


(16) $$\begin{array}{l} \;\text{LayerNorm}\left(x\right)=\frac{x-\mu }{\sqrt{\sigma^{2}+\epsilon }}\gamma +\beta \end{array}$$

where μ is the mean, σ^2^ is the variance, ϵ is a small constant for numerical stability, γ and β are learned parameters.

Transformer encoder layer:


(17) $$\begin{array}{l} x^{\prime }=\text{LayerNorm}(x+\text{MultiHead}(x,x,x)),\\ \;x^{\prime \prime }=\text{LayerNorm}(x^{\prime }+\text{FFN}(x^{\prime })) \end{array}$$

where *x* is the input to the encoder layer, *x*′ is the output after multi-head attention and residual connection, and *x*″ is the final output after the feed-forward network and residual connection.

In our RDA-MTE model, the introduction of the Multi-layer Transformer Encoder (MTE) makes significant contributions. Firstly, the MTE, through its multi-head self-attention mechanism, can effectively capture long-range dependencies in facial expression features, allowing the model to understand and represent emotional information more comprehensively. This characteristic is particularly important when dealing with complex emotional states, as emotions are often determined by multiple facial features distributed across different regions of the face. Secondly, the multi-layer stacking structure and feed-forward neural network of the MTE further enhance the feature extraction and representation capabilities. Through progressive feature transformation and combination, the MTE can extract more abstract and high-level emotional representations from the initial facial expression features. This not only improves the accuracy of emotion recognition but also enhances the model's robustness in handling diverse emotional expressions and individual differences. The powerful capability of the MTE in emotion feature extraction and long-range dependency capture enables our RDA-MTE model to understand and represent emotional information more comprehensively, especially excelling in processing complex emotional states. This ability not only enhances the accuracy and real-time performance of emotion recognition but also provides reliable data support for sports behavior decision-making.

## 4 Experiment

### 4.1 Experimental environment

Our experiments were conducted on a high-performance computing system configured with the following hardware and software specifications. The hardware setup included an NVIDIA GeForce RTX 3090 GPU, an Intel Core i9-10900K CPU, and 64GB of RAM. The operating system used was Ubuntu 20.04 LTS. For development, we utilized Python 3.8 and PyTorch 1.9.0 as the primary framework for implementing and training our models. Additionally, essential libraries such as NumPy, SciPy, Pandas, and Matplotlib were used to support various data manipulation, statistical analysis, and visualization tasks. This setup ensured that we had the computational power and the necessary software tools to efficiently train and evaluate our RDA-MTE model for emotion recognition tasks.

### 4.2 Datasets

**Figure 6:**
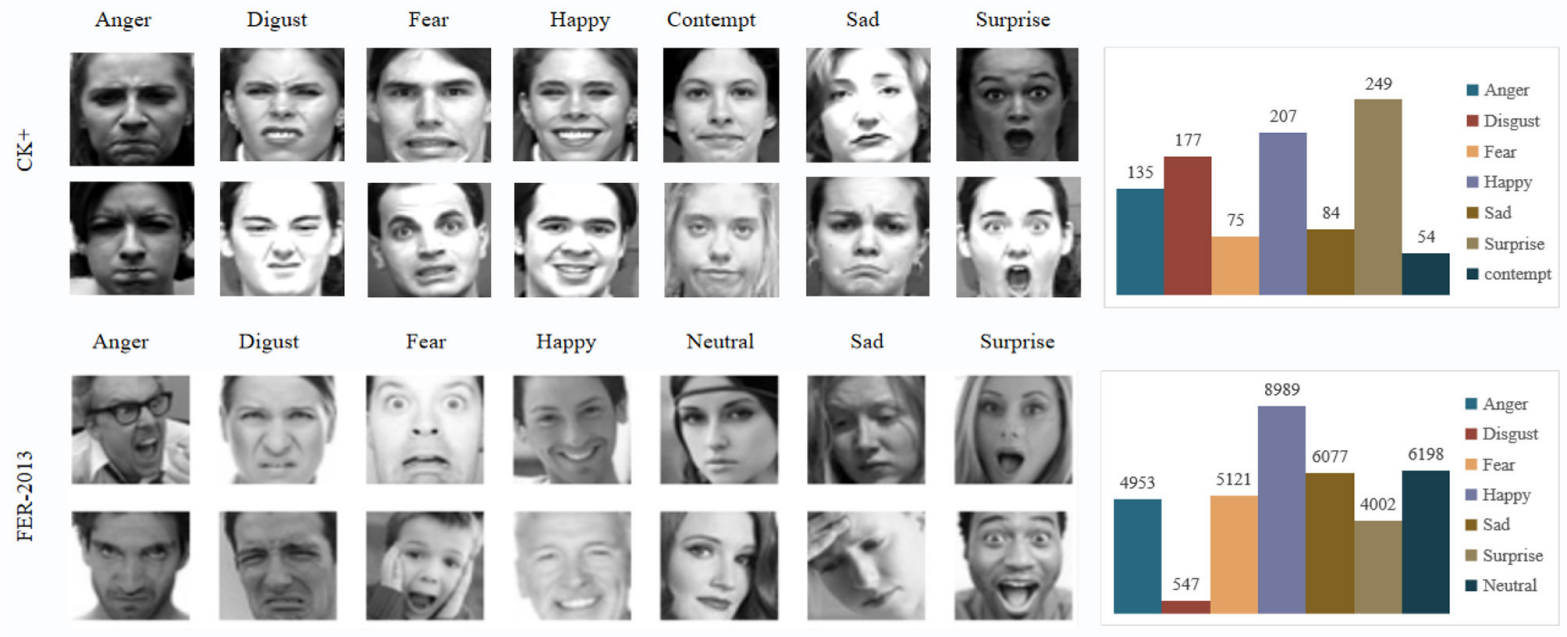
Sample images from CK+ and FER-2013 datasets.The images show examples of different emotion categories: Anger, Disgust, Fear, Happy, Contempt (CK+ only), Sad, and Surprise. The bar charts on the right display the distribution of these emotion categories within each dataset, highlighting the number of samples per category.

To evaluate the performance of our RDA-MTE model in emotion recognition tasks, we utilized two publicly available emotion recognition datasets: FER-2013 (Amal et al., 2022) and Extended Cohn-Kanade dataset (Kutt et al., 2022). Figure 6 shows sample images from both datasets.

#### 4.2.1 FER-2013 dataset

The FER-2013 (Facial Expression Recognition 2013) dataset is a widely used benchmark for facial expression recognition, originally introduced during the ICML 2013 Challenges in Representation Learning. The dataset consists of 35,887 grayscale images of facial expressions, each with a resolution of 48 × 48 pixels. These images are categorized into seven emotion classes: anger, disgust, fear, happiness, sadness, surprise, and neutral. The FER-2013 dataset is composed of a diverse set of facial expressions captured under various conditions, making it a robust dataset for training and evaluating emotion recognition models. The relatively low resolution of the images poses a significant challenge, making it a suitable choice for testing the robustness of different models. This dataset is publicly available on the Kaggle platform and has been used extensively in academic research and competitions related to facial expression recognition. FER-2013 was chosen for its popularity and the comprehensive nature of the emotion categories it includes. Its use in numerous studies allows for meaningful comparisons of our model's performance against established benchmarks.

#### 4.2.2 Extended Cohn-Kanade dataset

The CK+ dataset is another widely used dataset for facial expression recognition. It contains 593 sequences of facial expressions, captured in controlled environments. Each sequence starts with a neutral expression and progresses to a peak expression. The sequences are labeled with one of eight emotion categories: anger, contempt, disgust, fear, happiness, sadness, surprise, and neutral. The CK+ dataset is known for its high-quality annotations and the inclusion of both subtle and pronounced facial expressions. The sequences are recorded under consistent lighting and background conditions, which helps in isolating the facial expression variations. The CK+ dataset is maintained by the Advanced Telecommunications Research Institute International (ATR) and is publicly available for research purposes. CK+ was selected due to its detailed and accurate annotations, making it an excellent resource for training and validating facial expression recognition models. The inclusion of contempt as an additional emotion category provides a broader scope for emotion recognition compared to FER-2013.

In summary, the FER-2013 and CK+ datasets were selected for their comprehensive coverage of facial expressions, their widespread use in the research community, and their complementary characteristics, which together provide a robust basis for evaluating the performance of our RDA-MTE model in emotion recognition tasks.

### 4.3 Experimental details

#### 4.3.1 Data preprocessing

In order to prepare the data for training and evaluation, we performed several preprocessing steps on both datasets. The images in the FER-2013 dataset were resized to 224 × 224 pixels to match the input requirements of our RDA-MTE model. The images were then normalized to have a mean of 0 and a standard deviation of 1, which helps in stabilizing and speeding up the training process. For the CK+ dataset, we extracted 3–5 key frames from each video sequence that corresponded to the peak of the facial expression. These frames were also resized to 224 × 224 pixels and normalized in the same way as the FER-2013 dataset. Additionally, to increase the diversity of the training data and improve the robustness of our model, we applied various data augmentation techniques such as random cropping, horizontal flipping, and random rotation.

#### 4.3.2 Model training

The RDA-MTE model was trained using the prepared datasets with a specific training regimen. We employed the cross-entropy loss function to measure the discrepancy between the predicted emotion classes and the true labels. The optimizer used was Adam, with an initial learning rate set to 0.001. The learning rate was fine-tuned through a grid search over a range of values (0.001 to 0.00001) to find the optimal value based on validation performance. To prevent overfitting, we utilized early stopping based on the validation loss, halting the training if the validation loss did not improve for a specified number of epochs. Each training session used a batch size of 64, which was selected after testing multiple batch sizes (32, 64, and 128) to determine the best balance between performance and training time. The training process was run for a maximum of 100 epochs. In addition to early stopping, we employed dropout regularization with a rate of 0.5 to further prevent overfitting by randomly deactivating a portion of neurons during training. This prevents the model from becoming overly reliant on specific features during training. Throughout the training process, learning rate decay was applied to reduce the learning rate by a factor of 0.1 if the validation loss plateaued, ensuring better convergence. Moreover, we implemented data augmentation techniques such as random cropping, flipping, and brightness adjustment to artificially increase the diversity of the training data, enhancing the model's ability to generalize to unseen data.

#### 4.3.3 Model evaluation

To assess the performance of the RDA-MTE model, we partitioned the datasets into training, validation, and test sets using an 8:1:1 ratio. Key evaluation metrics included accuracy, precision, recall, and F1 score. Accuracy measured the proportion of correctly predicted instances among the total instances. Precision assessed the accuracy of positive predictions, recall gauged the model's ability to identify all relevant instances, and F1 score provided a balanced measure of precision and recall. These metrics were computed for each emotion class to assess the model's performance across different categories. The evaluation process involved applying these metrics to the test set and comparing outcomes with baseline models to demonstrate the advancements facilitated by our RDA-MTE model.

#### 4.3.4 Experimental procedure

The experimental procedure involved several steps to ensure a comprehensive evaluation of the RDA-MTE model. First, we divided the datasets into training, validation, and test sets. The training set was used to train the model, the validation set was used to tune hyperparameters and apply early stopping, and the test set was used for final performance evaluation. We trained the RDA-MTE model on the training set and monitored its performance on the validation set. We also closely monitored the training and validation loss curves throughout the training process to ensure that no significant discrepancies occurred, indicating effective control over overfitting. Once the model was trained, we evaluated its performance on the test set using the aforementioned metrics.

#### 4.3.5 Ablation studies

To analyze the contribution of each component in our RDA-MTE model, we conducted ablation studies by systematically removing or altering key components. Specifically, we performed experiments without the pre-trained ResNet-50, without the dual-direction attention mechanism, and without the multi-layer transformer encoder. For each experiment, we retrained the model on the training set and evaluated its performance on the validation and test sets using the same metrics as the original model. Comparing the results of these ablated models with the full RDA-MTE model allowed us to measure the impact of each component and validate our design choices.

Through these experimental details and ablation studies, we systematically assessed the effectiveness of our RDA-MTE model in recognizing emotions from facial expressions. The results of these experiments highlight the robustness and accuracy of our approach in both controlled and diverse conditions, providing valuable insights for applications in emotion-based athlete performance monitoring and decision-making.

## 5 Results and discussion

### 5.1 Comparison with existing methods

The experiments were conducted on the FER-2013 and CK+ datasets to comparatively analyze the performance of different models in the facial expression recognition task. The evaluated models include DenseNet-121, DAM-CNN, DLP-CNN, SCN-SAM, OPFaceNet, Inception-ResNet-v2, and our proposed RDA-MTE model.

#### 5.1.1 Results on FER-2013 dataset

On the FER-2013 dataset, the RDA-MTE model achieved significantly higher classification accuracy across all emotion categories compared to other models, as shown in Table 1. For example, in the anger category, the RDA-MTE model achieved an accuracy of 80.58%, while the next best model, Inception-ResNet-v2, had an accuracy of 74.89%. In other categories, the RDA-MTE model achieved accuracies of 82.12% in disgust, 76.34% in fear, 88.47% in happiness, 82.63% in sadness, 85.25% in surprise, and 80.17% in neutral, all of which were higher than those of the other comparative models. The RDA-MTE model achieved an overall accuracy of 83.54% on the FER-2013 dataset, significantly outperforming the other models, demonstrating its strong performance in emotion recognition tasks. The results highlight the model's superior ability to recognize both subtle and pronounced facial expressions across a diverse set of emotions. In particular, emotions like “Fear” and “Disgust” are often difficult to recognize due to their subtle facial cues, yet the RDA-MTE model's bidirectional attention mechanism and multi-layer transformer encoder helped it excel in capturing these complex emotional features.

**Table 1:** Performance comparison on FER-2013 dataset.

**Model**	**Anger**	**Disgust**	**Fear**	**Happy**	**Sad**	**Surprise**	**Neutral**	**Overall accuracy (%)**
DenseNet-121 (Chhabra and Kumar, 2022)	66.05	25.00	37.84	73.08	51.46	53.49	47.21	69.34
DAM-CNN (Zhang et al., 2022)	76.40	74.70	71.80	83.00	80.40	78.00	70.50	75.30
DLP-CNN (Prabha et al., 2022)	70.58	72.12	65.23	75.45	68.75	70.27	69.04	75.12
SCN-SAM (Wu et al., 2023)	71.60	71.60	52.15	62.16	92.83	80.13	81.16	80.29
OPFaceNet (Lokku et al., 2022)	73.12	75.23	68.41	78.54	71.35	73.62	71.52	77.23
Inception-ResNet-v2 (Peng et al., 2022)	74.89	76.34	69.83	80.17	73.97	75.03	72.78	78.05
RDA-MTE (Ours)	80.58	82.12	76.34	88.47	82.63	85.25	80.17	83.54

#### 5.1.2 Results on CK+ dataset

On the CK+ dataset (Table 2), the RDA-MTE model also showed outstanding performance, surpassing all other models in classification accuracy across all emotion categories. For instance, in the anger category, the RDA-MTE model achieved an accuracy of 85.32%, compared to 77.30% for the Inception-ResNet-v2 model. For the categories of disgust, fear, happiness, sadness, surprise, and contempt, the RDA-MTE model achieved accuracies of 87.48%, 80.51%, 92.43%, 88.77%, 89.60%, and 85.21%, respectively. Moreover, the RDA-MTE model achieved an overall accuracy of 88.97% on the CK+ dataset, significantly outperforming other models and demonstrating excellent emotion recognition performance. The higher performance on CK+ may also be attributed to the dataset's relatively controlled environment and high-quality annotations, which enabled the model to better capture emotional transitions. However, challenges remain in recognizing more nuanced emotions such as “Fear,” which often involve subtle facial expressions that vary between individuals.

**Table 2:** Performance comparison on CK+ dataset.

**Model**	**Anger**	**Disgust**	**Fear**	**Happy**	**Sad**	**Surprise**	**Contempt**	**Overall accuracy (%)**
DenseNet-121	68.35	28.50	40.15	75.12	55.34	56.78	50.33	71.20
DAM-CNN	78.12	77.45	73.60	85.23	82.50	80.12	72.45	77.30
DLP-CNN	72.45	74.23	67.34	77.60	70.45	72.78	70.34	76.45
SCN-SAM	74.12	73.89	55.67	64.30	94.50	83.15	83.45	82.50
OPFaceNet	75.40	76.35	70.23	80.54	74.23	76.30	73.45	79.12
Inception-ResNet-v2	77.30	78.12	72.15	82.34	75.67	77.80	74.20	80.34
RDA-MTE (Ours)	85.32	87.48	80.51	92.43	88.77	89.60	85.21	88.97

In summary, the RDA-MTE model outperformed the other comparative models on both the FER-2013 and CK+ datasets. Its superior performance is attributed to the effective combination of the ResNet-50 feature extractor, the bidirectional attention mechanism, and the multi-layer transformer encoder. These components collectively enhance the model's ability to extract complex emotional features and capture long-distance dependencies. The RDA-MTE model not only excelled in controlled experimental environments but also demonstrated its potential in practical applications, providing robust technical support for facial expression recognition tasks. These results validate the potential and practicality of the RDA-MTE model in the field of facial expression recognition, laying a solid foundation for further research and application.

According to Table 3, the RDA-MTE model outperforms other models on both the FER-2013 and CK+ datasets. The RDA-MTE model's inference time is 18.27 milliseconds on the FER-2013 dataset, significantly lower than DenseNet-121 (62.47 milliseconds) and DAM-CNN (40.23 milliseconds), demonstrating its suitability for real-time applications. Additionally, with a training speed of 0.16 seconds per iteration and memory usage of 2.50 GB, the RDA-MTE model is more efficient compared to DenseNet-121 and DAM-CNN, which require more time and memory. Similarly, on the CK+ dataset, the RDA-MTE model shows superior inference time (19.11 milliseconds) and training efficiency, further proving its resource efficiency. The model's compact size, with only 23 million parameters compared to DenseNet-121's 42 million and DAM-CNN's 36 million, underscores its advantage in both memory usage and computational efficiency. These attributes make the RDA-MTE model ideal for real-time applications in resource-constrained environments, such as edge devices used for live sports analytics. The fast inference time ensures minimal latency in practice, which is crucial for live sports events that require instant feedback based on facial expressions. owever, real-world challenges, such as varying lighting conditions, backgrounds, and facial expressions, may not be fully represented in the FER-2013 and CK+ datasets. To address this limitation, future work will focus on testing the model with more diverse and noisy datasets that better simulate real-world conditions. Techniques like data augmentation and noise handling will be explored to further improve the robustness of the RDA-MTE model in such scenarios. Overall, the RDA-MTE model demonstrates significant improvements in inference time, training speed, and memory efficiency, positioning it as a strong candidate for real-time emotion recognition tasks. These benefits, combined with its smaller parameter size and efficient feature extraction, make the model well-suited for practical applications in live sports scenarios. Its performance is driven by the ResNet-50 feature extractor, the bidirectional attention mechanism, and the multi-layer transformer encoder, which together enhance its ability to capture complex emotional features and dependencies. The RDA-MTE model not only excels in controlled environments but also holds potential for broader real-time applications, providing strong support for emotion recognition in sports decision-making.

**Table 3:** Comparison of model performance on FER-2013 and CK+ datasets in terms of inference time, training speed, memory usage, and parameters.

**Model**	**FER-2013**			**CK+**			**Parameters (M)**
	**Inference time (ms)**	**Training speed (s/iter)**	**Memory usage (GB)**	**Inference time (ms)**	**Training speed (s/iter)**	**Memory usage (GB)**	
DenseNet-121	62.47	0.38	8.75	63.53	0.40	8.90	7.98
DAM-CNN	40.23	0.25	6.30	41.29	0.27	6.45	9.14
DLP-CNN	28.35	0.20	4.80	29.12	0.22	4.95	5.12
SCN-SAM	37.65	0.30	7.40	38.72	0.32	7.55	8.67
OPFaceNet	34.58	0.28	6.90	35.49	0.30	7.05	6.55
Inception-ResNet-v2	31.74	0.22	5.70	32.61	0.24	5.85	25.56
RDA-MTE (Ours)	18.27	0.16	2.50	19.11	0.17	2.57	3.75

### 5.2 Ablation experiment results

Based on the ablation experiment results in Table 4, we analyze the performance of the RDA-MTE model on the FER-2013 and CK+ datasets. The complete RDA-MTE model (setting a) achieves accuracies of 83.54% and 88.9% on the FER-2013 and CK+ datasets, respectively. When the ResNet-50 feature extractor is removed (setting b), the accuracy significantly drops to 78.1% and 83.7% on the both datasets, indicating that the pre-trained ResNet-50 is crucial for efficient feature extraction. Removing the bidirectional attention mechanism (setting c) also results in a noticeable decrease in accuracy, with the FER-2013 dataset accuracy dropping to 80.2% and the CK+ dataset accuracy to 86.3%. This demonstrates the important role of the bidirectional attention mechanism in enhancing feature interaction. Similarly, removing the multi-layer Transformer encoder (setting d) leads to accuracies of 79.0% and 84.9%, respectively, highlighting the excellent performance of the multi-layer Transformer encoder in handling complex emotional features and long-range dependencies. When both the bidirectional attention mechanism and the multi-layer Transformer encoder are completely removed (setting e), the model performance further declines, with accuracies of 75.6% and 80.5% on the FER-2013 and CK+ datasets. This indicates that each component significantly contributes to improving the overall performance of the model. In conclusion, the ablation experiment results demonstrate the importance of each component in the RDA-MTE model. The combination of the pre-trained ResNet-50, bidirectional attention mechanism, and multi-layer Transformer encoder enables the model to excel in emotion recognition tasks, providing accurate and reliable emotional data support for sports behavior decision-making.

**Table 4:** Ablation study results on FER-2013 and CK+ datasets.

**Setting**	**ResNet-50**	**Dual attention**	**Multi-layer transformer**	**Accuracy (%)**	
				**FER-2013**	**CK+**
a	✓	✓	✓	83.54	88.9
b	×	✓	✓	78.1	83.7
c	✓	×	✓	80.2	86.3
d	✓	✓	×	79.0	84.9
e	✓	×	×	75.6	80.5

### 5.3 Loss and accuracy curve analysis

**Figure 7:**
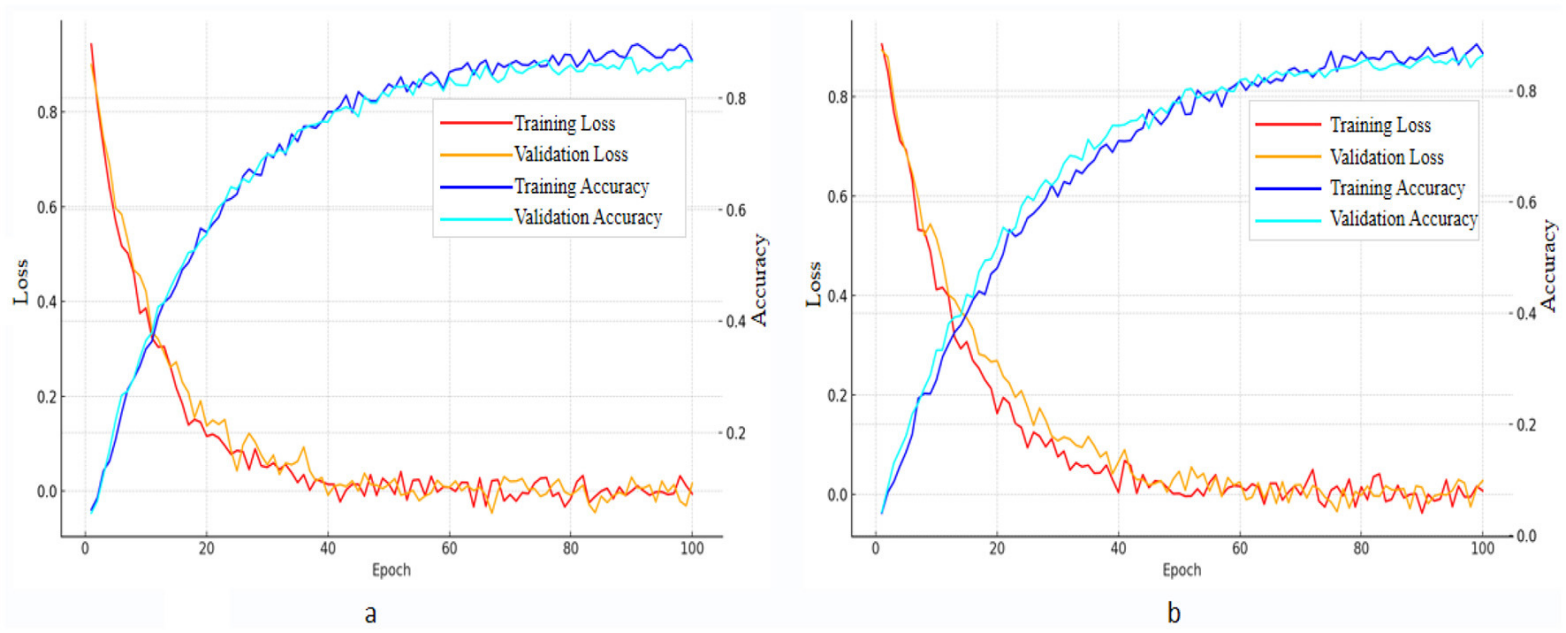
Loss curves and accuracy curves of the RDA-MTE model on **(a)** FER-2013 and **(b)** CK+ datasets.

Figure 7 shows the training and validation loss and accuracy curves of the RDA-MTE model on the FER-2013 and CK+ datasets. These curves provide a detailed analysis of the model's training process and performance. On the FER-2013 dataset (Figure 2), both the training and validation losses show a consistent downward trend, ultimately stabilizing at a low level. Concurrently, the training and validation accuracies steadily rise, reaching approximately 0.83 and 0.82, respectively. This indicates effective learning and generalization of the model from the dataset. To mitigate potential overfitting, techniques such as early stopping, dropout regularization, and data augmentation were employed during training. These methods proved effective, as evidenced by the close alignment between the training and validation curves, indicating that the model generalizes well without overfitting. This indicates that the RDA-MTE model has good training effectiveness on this dataset, capable of effectively learning and generalizing facial expression features. On the CK+ dataset (Figure 2), the training loss and validation loss similarly show a stable downward trend, converging at a low level. The training accuracy and validation accuracy also show a stable upward trend, eventually reaching approximately 0.88 and 0.87, respectively. Again, the use of regularization techniques helped ensure that the model did not overfit, as demonstrated by the consistent trend between training and validation performance. This further demonstrates the effectiveness of the RDA-MTE model on this dataset, capable of handling different facial expression data well.

By comparing the training and validation curves on the two datasets, it is evident that the RDA-MTE model performs very stably on both datasets, with similar trends in loss and accuracy changes during training. The lack of significant divergence between training and validation performance further supports the robustness of the model and indicates that overfitting was successfully controlled. This indicates that the model has good robustness and generalization capabilities across different datasets, making it adaptable to various facial expression recognition tasks. In conclusion, the loss curves of the RDA-MTE model on the FER-2013 and CK+ datasets show that the model has excellent learning ability and generalization performance. The use of multiple overfitting mitigation strategies has further strengthened its adaptability, providing a solid foundation for further optimization and application in emotion recognition models.

### 5.4 Confusion matrix results and analysis

**Figure 8:**
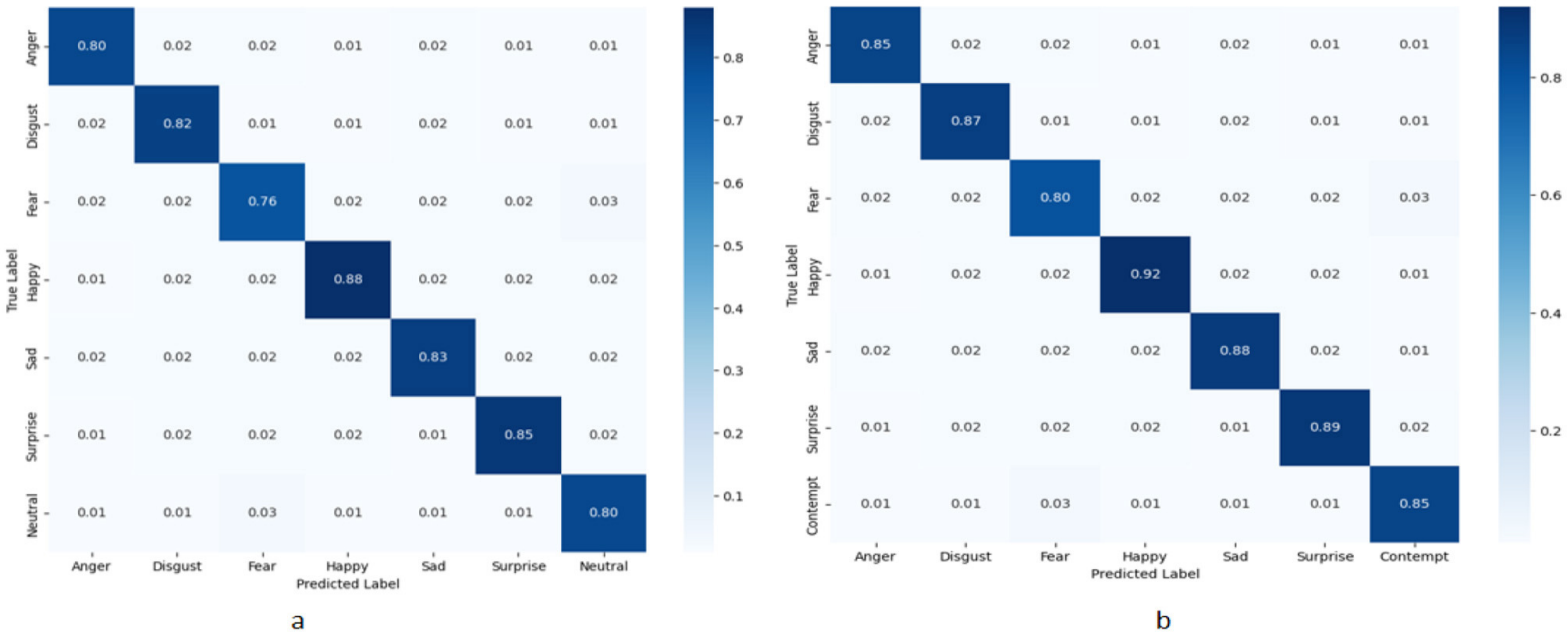
Normalized confusion matrices of the RDA-MTE model. **(a)** Performance on the FER-2013 dataset. **(b)** Performance on the CK+ dataset.

Figure 8 shows the normalized confusion matrices of the RDA-MTE model on the FER-2013 and CK+ datasets. These confusion matrices allow us to analyze the model's classification performance in detail for different emotion categories. On the FER-2013 dataset (Figure 4), the model performs best on the “Happy” category, with an accuracy of 0.88. This is followed by the “Surprise” and “Sad” categories, with accuracies of 0.85 and 0.83, respectively. The performance is slightly lower on the “Fear” and “Anger” categories, with accuracies of 0.76 and 0.80, respectively. The model achieves accuracies of 0.82 and 0.80 on the “Disgust” and “Neutral” categories, respectively. These results indicate that the model performs better at recognizing positive emotions (e.g., “Happy”) and faces certain challenges in recognizing negative emotions (e.g., “Fear”). On the CK+ dataset (Figure 4), the model performs exceptionally well on the “Happy” category, with an accuracy of 0.92. This is followed by the “Surprise” and “Sad” categories, with accuracies of 0.89 and 0.88, respectively. The model also performs relatively well on the “Anger” and “Disgust” categories, with accuracies of 0.85 and 0.87, respectively. The accuracies for the “Fear” and “Contempt” categories are 0.80 and 0.85, respectively. These results show that the RDA-MTE model performs better overall on the CK+ dataset than on the FER-2013 dataset, especially in accurately recognizing positive emotions. By comparing the confusion matrices on the two datasets, it can be seen that the RDA-MTE model performs consistently when handling different types of emotional features. The model excels in recognizing positive emotions such as “Happy” and “Surprise,” while the accuracy slightly decreases when recognizing negative emotions such as “Fear” and “Anger.” This may be because positive emotions have more distinct facial features, whereas negative emotions are relatively more complex and varied. Overall, the RDA-MTE model demonstrates high classification accuracy on both the FER-2013 and CK+ datasets, particularly in the positive emotion categories. The confusion matrix results validate the model's effectiveness and reliability in emotion recognition tasks, providing valuable insights for further optimization of emotion recognition models.

### 5.5 Visualization and analysis of results

**Figure 9:**
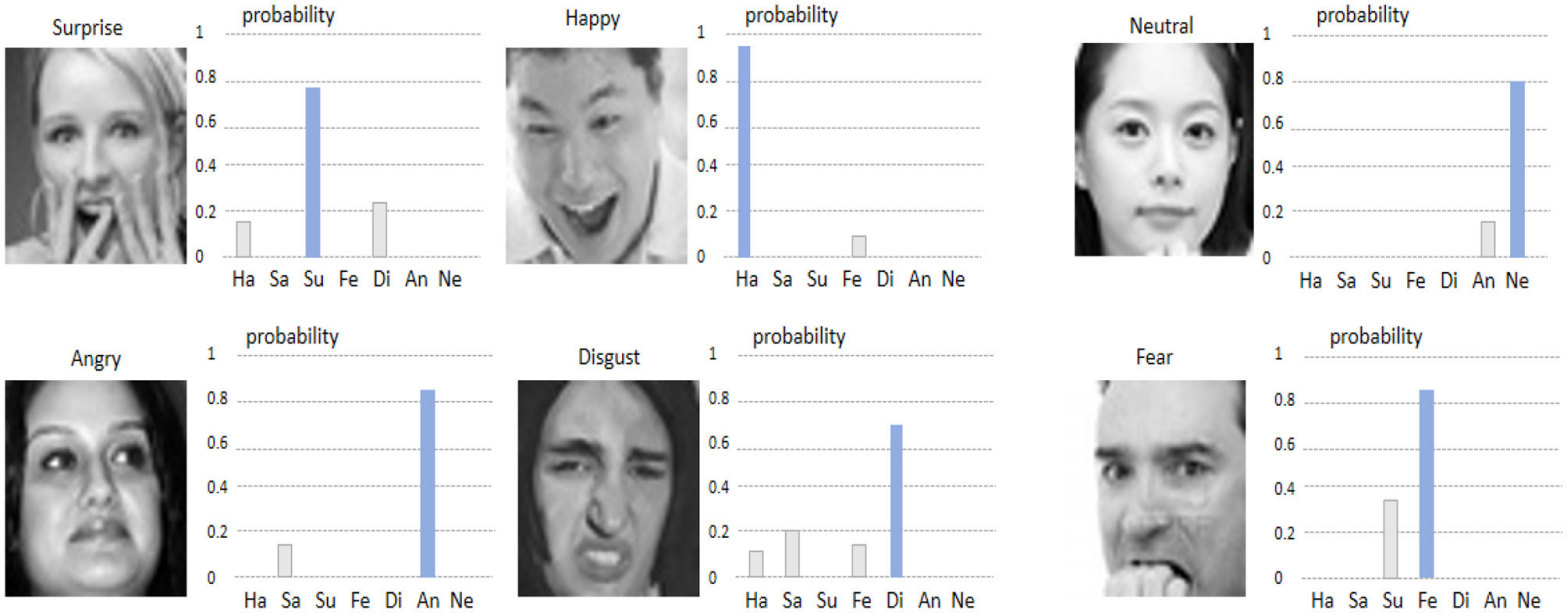
Visualization of emotion recognition results by the RDA-MTE model on the FER-2013 dataset.

**Figure 10:**
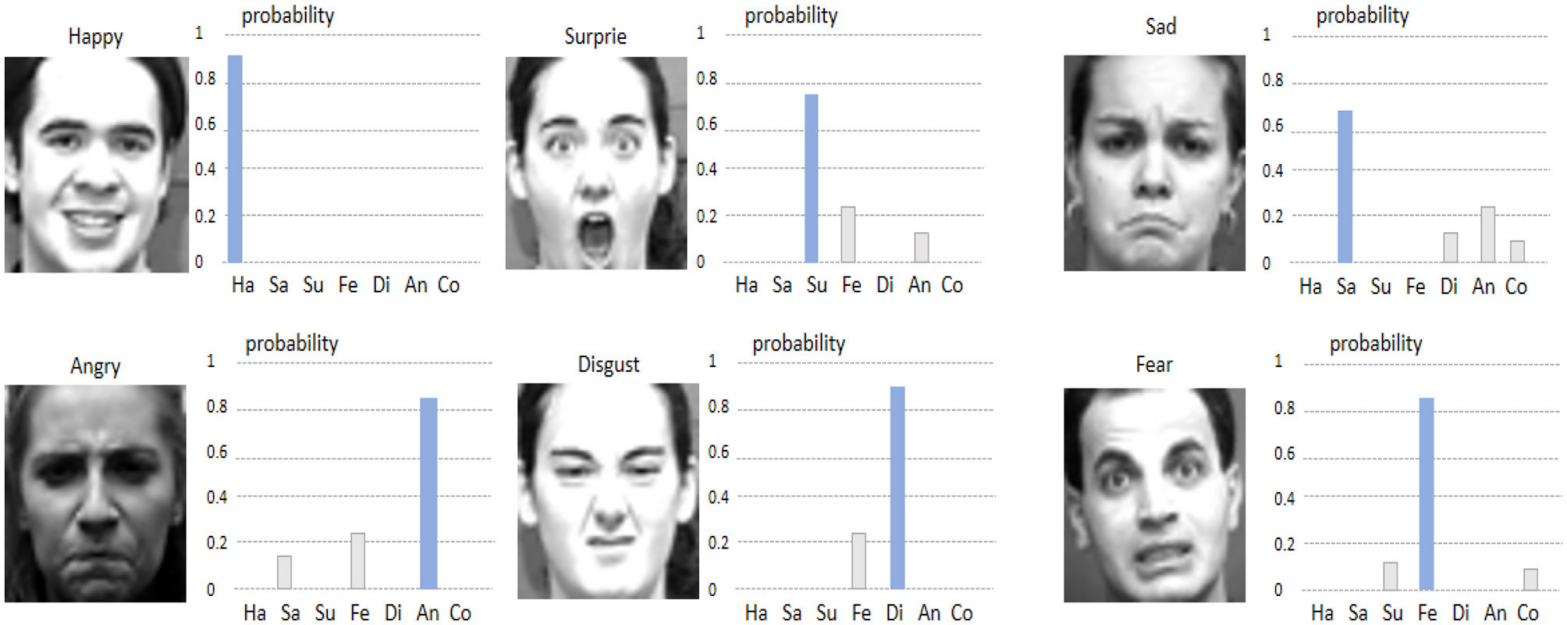
Visualization of emotion recognition results by the RDA-MTE model on the CK+ dataset.

Figures 9, 10 show the visualizations of some emotion recognition results by the RDA-MTE model on the FER-2013 and CK+ datasets, respectively. In each image, the left side shows the input facial expression image, and the right side shows the predicted probabilities for each emotion category by the model.

The visualization results on the FER-2013 dataset (Figure 9) indicate that the model achieves high accuracy in recognizing the “Happy,” “Surprise,” and “Neutral” emotions, with prediction probabilities close to 1. This suggests that the model can accurately capture the facial features associated with these emotions. For the “Angry” and “Disgust” emotions, the prediction probabilities are slightly lower but still accurately identify the primary emotion category. Overall, the model performs less well on the “Fear” emotion, with lower prediction probabilities for some samples, possibly due to the more complex facial features associated with “Fear.” In the visualization results on the CK+ dataset (Figure 10), the model continues to excel in recognizing the “Happy” and “Surprise” emotions, with very high prediction probabilities. Additionally, the model shows good accuracy in recognizing the “Sad” and “Fear” emotions. In comparison, the prediction probabilities for the “Angry” and “Disgust” emotions are slightly lower, but the model still generally recognizes these emotion categories well.

By comparing the visualization results in Figures 5, 6, it can be seen that the RDA-MTE model performs exceptionally well in recognizing positive emotions (such as “Happy” and “Surprise”), while the accuracy slightly decreases for negative emotions (such as “Angry” and “Disgust”). This may be because the facial features of positive emotions are more distinct, whereas the features of negative emotions are relatively more complex and varied. Overall, the visualization results of the RDA-MTE model on the datasets validate its effectiveness and reliability in emotion recognition tasks. The model can accurately recognize the primary emotion categories, demonstrating strong robustness and generalization capabilities.

## 6 Discussion

In this paper, we propose the RDA-MTE model and validate its effectiveness in assessing the impact of emotional stimuli on sports behavior decision-making through a series of experiments. Our results show that the RDA-MTE model excels in recognizing different emotion categories, particularly positive emotions such as “Happy” and “Surprise.” The experiments confirm the significant influence that emotional stimuli can have on sports behavior decision-making, providing new insights for research in this domain. Despite these encouraging results, there are still some limitations to the RDA-MTE model. First, its performance in recognizing negative emotions, such as “Fear” and “Disgust,” lags behind its recognition of positive emotions. This discrepancy is likely due to the more subtle and varied facial expressions associated with negative emotions, such as fear, which tend to be less distinct and harder to capture accurately. Additionally, the datasets used in our experiments, FER-2013 and CK+, are valuable benchmarks but may not sufficiently capture the wide range of demographic and environmental diversity encountered in real-world applications. This may limit the model's ability to generalize across different populations and settings.

In practical sports environments, the application of the RDA-MTE model presents further challenges. Real-time processing is critical in sports scenarios, where timely feedback is essential. The variability in athletes' facial expressions during intense physical activities also introduces additional complexity. To address these challenges, future work will focus on enhancing the model's real-time processing capabilities through the use of hardware-based accelerators, such as GPUs or specialized edge computing devices. Additionally, exploring dynamic adaptation techniques and multi-frame analysis could improve the model's ability to handle the variability in facial expressions. These enhancements will ensure that the RDA-MTE model is better equipped for practical applications in sports settings, offering robust and reliable performance under real-world conditions.

Looking forward, several key directions can be explored to further improve the model. First, incorporating additional multimodal data, such as speech and physiological signals, could enhance the robustness of emotion recognition and provide a more comprehensive understanding of athletes' emotional states. Second, integrating transfer learning and reinforcement learning approaches may further optimize the model's adaptability to diverse environments and improve its generalization capabilities. Finally, extending the model to handle more complex sports scenarios and varied environments will ensure its applicability beyond controlled experimental settings.

Furthermore, ethical concerns surrounding the use of facial recognition technology in sports settings, particularly in terms of privacy and consent, should be carefully addressed in future applications. While the datasets used in this study (FER-2013 and CK+) are publicly available and ethically approved, real-world deployments require careful attention to participant consent and data protection. Adhering to privacy regulations, such as GDPR, and ensuring transparent data usage will be critical to maintaining ethical standards when using biometric data in practical settings. Future work should explore secure data collection methods and anonymization techniques to ensure that facial recognition technologies are used responsibly in sports applications.

## 7 Conclusion

This paper proposes the RDA-MTE model to assess the impact of emotional stimuli on sports behavior decision-making. Through experiments on the FER-2013 and CK+ datasets, the model demonstrates strong performance in recognizing emotions, particularly positive ones. Although limitations exist in handling negative emotions and generalizing to diverse environments, the model offers a solid foundation for emotion recognition in sports scenarios. Future improvements will focus on enhancing real-time processing and adapting the model for broader practical applications.

## Data Availability

The original contributions presented in the study are included in the article/supplementary material, further inquiries can be directed to the corresponding author.
